# Racial Differences in Length of Stay After Atrial Fibrillation Ablation

**DOI:** 10.1111/pace.70029

**Published:** 2025-08-21

**Authors:** Waseem Nosair, Yasmina Sirgi, Evan Czulada, Nebu Alexander, Jamal Smith, Sarahfaye Dolman, Apostolos Tsimploulis, Athanasios Thomaides, David Strouse, Zayd Eldadah, Sung Lee, Morgana Mongraw‐Chaffin, William S. Weintraub

**Affiliations:** ^1^ School of Medicine Georgetown University Washington District of Columbia USA; ^2^ Department of Medicine MedStar Washington Hospital Center Washington District of Columbia USA; ^3^ Population Health Research MedStar Health Research Institute MedStar Washington Hospital Center Washington District of Columbia USA; ^4^ Section of Electrophysiology MedStar Heart and Vascular Institute Washington Hospital Center Washington District of Columbia USA

**Keywords:** atrial fibrillation, catheter ablation, gender disparities, health equity, quality outcomes, racial disparities

## Abstract

**Background:**

Racial and sex disparities in catheter ablation (CA) utilization for atrial fibrillation (AF) have been documented. Less is known about race and sex differences in comorbidity burden and quality of care outcomes after CA.

**Objectives:**

We sought to characterize racial and sex differences in patient and procedural characteristics and their impact on prolonged length of stay (LOS) after CA for AF.

**Methods:**

A retrospective cohort of patients that received CA for AF between 2018 and 2024 was developed from our single center NCDR registry. The analysis was restricted to non‐Hispanic White (NHW) and non‐Hispanic Black (NHB) patients due to small sample sizes for other groups. The association between race, sex, and prolonged LOS was evaluated using a multivariable stepwise regression model. A sensitivity analysis was performed with the composite outcome of complications given its sparsity. Causal mediation analysis was performed to assess whether race differences in prolonged LOS were mediated by complications.

**Results:**

Between 2018 and 2024, 3345 patients were included in the cohort. NHB patients were younger and more likely to have Medicaid insurance, higher BMI, higher comorbidity burden, history of atrial flutter, prolonged LOS, and complications after ablation. They were less likely to have prior CA. Female patients were older, less likely to have private insurance and prior CA, and more likely to have paroxysmal AF, transient ischemic attack, and chronic lung disease. NHB race [aOR 1.90 (95% CI: 1.24–2.88), *p* = 0.003] and a composite outcome of procedural complications [aOR 156 (95% CI: 72.5–377), *p* < 0.001], but not sex, were independently associated with prolonged LOS. The effect of race on prolonged LOS was partially mediated by higher comorbidity burden and obesity among NHB, but not by complications, Medicaid status, or AF type.

**Conclusion:**

Prolonged LOS was more frequent among NHB patients undergoing CA. The higher odds of prolonged LOS among NHB patients was not mediated by a higher incidence of complications, suggesting that other factors like comorbidity burden and social determinants of health (SDOH) are more significant contributors. Policies focused on improving comorbidity management and addressing sociocultural disparities may reduce prolonged hospitalizations after CA for AF.

**Abbreviations**
AF: atrial fibrillation
BMI: body mass index
CA: catheter ablation
CI: confidence interval
LOS: length of stay
NCDR: National Cardiovascular Data Registry
NHB: non‐Hispanic Black
NHW: non‐Hispanic White
OR: odds ratio

## 1 Introduction

Race and sex disparities have been documented across the spectrum of AF care. While non‐Hispanic Black (NHB) patients have a higher prevalence of traditional risk factors for AF, they are less likely to be diagnosed with AF compared with non‐Hispanic White (NHW) patients [1]. Despite a lower prevalence of AF in NHB patients, NHB patients with AF are at an increased risk of stroke, heart failure, and all‐cause mortality compared to NHW patients, which has been termed the AF paradox [2, 3]. It is unclear whether these trends represent a true paradox or if it may be explained by ascertainment bias from underdiagnosis or delayed diagnosis. Differences in risk factors may partially explain ascertainment bias, as approximately 50% of the AF burden among NHB patients was not accounted for by traditional risk factors in one cohort [4]. This suggests the presence of additional novel risk factors in this population [4]. Beyond differences in diagnosis, minority groups including female patients are less likely to receive access to specialty care once diagnosed with AF, including lower utilization of rhythm control strategies, higher complication rates after catheter ablation, and worse outcomes [5, 6].

Less is known about racial and sex differences in outcomes after ablation including length of stay (LOS). LOS after CA is an important quality metric from a patient and health systems perspective as it may reflect better patient satisfaction, outcomes, and lower cost and resource utilization. Same day discharge after ablation has been shown to be as safe and effective as overnight observation [7]. Identifying disparities in LOS affecting minority groups is crucial for ensuring equitable care. We sought to characterize race and sex differences in patient characteristics, complications, and LOS after CA at an urban academic center serving a diverse patient population. We hypothesized that non‐procedural factors may have a larger effect on prolonged LOS. We adopted a “residual direct effect approach,” which estimates race disparity as the remaining difference between groups after adjusting for other covariates [8]. We distinguish between health differences and disparities. Differences may be acceptable and justified if they reflect variation in health status or preferences between groups, whereas disparities reflect unjust differences in care delivery that are not explained by underlying health needs or preferences [9].

## 2 Methods

We conducted a retrospective cohort study of patients that underwent CA for AF between 2018 and 2024. Information was collected according to the standards of the National Cardiovascular Data Registry (NCDR) AFib Ablation Registry. A prespecified data collection form was completed for each patient undergoing CA. Data quality was ensured through internal verification of the NCDR AFib Ablation Registry's guidelines, which outline the data reporting process and supervised by a steering committee with periodic maintenance updates [10]. Patients with missing values for key covariates and outcomes were excluded.

Our primary outcome was prolonged LOS, defined as a LOS greater than 1 day. The primary exposure variables were self‐reported race and sex. While race is a social construct, racial identity has been shown to be a strong predictor of health outcomes. The NCDR registry uses self‐reported race to organize individuals into NHB and NHW categories, as it is preferred over observer classification [11]. The analysis was restricted to NHW and NHB patients due to limited data on other race groups.

Baseline characteristics and outcomes were also stratified by race and sex. Continuous variables were summarized using medians and interquartile ranges (IQR) and compared using the Wilcoxon rank‐sum test for non‐normally distributed data. Categorical variables were presented as frequencies and percentages and compared with Pearson's Chi‐squared test or Fisher's exact test for variables with small cell counts. A *p* value of less than 0.05 was considered statistically significant for all tests. All statistical tests were performed with R [12].

Univariable logistic regression was performed to assess the relationship between each predictor variable and the outcome prolonged LOS. An epidemiologic approach was used to select predictors for the multivariable logistic regression models based on predictors of LOS described in the literature and clinical judgment. We assessed the independent effect of race on prolonged LOS using a multivariable logistic regression model adjusting for insurance status, comorbidity burden, AF characteristics, atrial flutter history, procedural characteristics, and complications. To optimize the model, a stepwise algorithm was performed based on the Akaike Information Criterion (AIC) using the stepAIC function from the MASS package allowing for both forward and backward selection of predictors that are not already established [13]. The model with the lowest AIC was retained. Multicollinearity among predictors was assessed by calculating the variance inflation factor (VIF). A sensitivity analysis was conducted by fitting logistic regression models with and without complications to assess this rare predictor's impact on the model's performance.

To explore potential pathways underlying the association between race and prolonged LOS, we conducted causal mediation analysis using the mediation package in R [14]. A model‐based approach was used to decompose the race–LOS association into direct and indirect components based on methods described by Imai et al. [15]. In this framework, the total effect of Black race on prolonged LOS is partitioned into a direct effect (the portion not through the mediator) and an indirect (mediated) effect. For each candidate mediator (complications, Medicaid insurance, modified CCI, obesity, atrial flutter, or long‐standing persistent AF), we fit two regression models: a mediator model predicting the mediator from race, adjusting for other covariates, and an outcome model predicting LOS from race, the mediator, and covariates. Using both models, the mediation function estimates the average causal mediation effect (ACME) (the indirect effect via the mediator) and the average direct effect (ADE) (the remaining effect of race on LOS). The ACME quantifies how much of the total race–LOS association is carried through that mediator, while the ADE is the residual race effect after accounting for the mediator. The sum of ACME and ADE equals the total effect, and the proportion mediated (ACME/total) represents the fraction of the total effect explained by the mediator. Estimates and confidence intervals for ACME, ADE, and the total effect were obtained using nonparametric bootstrapping with 1000 simulations.

This study was approved by the MedStar institutional review board (IRB ID: STUDY00008324).

## 3 Results

This study represents a cohort of 3345 patients from our single center NCDR registry who underwent CA between 2018 and 2024. Baseline clinical characteristics are reported stratified by race (Table 1) and sex (Table 2). Patients undergoing CA were mostly male and NHW, compared to female and NHB. NHB patients and males were typically younger at time of diagnosis compared with NHW patients and females. Insurance coverage varies notably, with more NHB patients and females enrolled in Medicaid and fewer in Medicare. Males and NHB patients were more likely to have private insurance than females and NHW patients. NHB patients and females had a higher BMI than NHW patients and males.

**TABLE 1:** Patient demographics by self‐reported race.

Characteristic	NHW patients, *n* = 2829^a^	NHB patients, *n* = 516^a^	*p* value^b^
Age	67 (60, 74)	65 (55, 71)	**<0.001**
Sex			**<0.001**
Male	1866 (66%)	296 (57%)	
Female	963 (34%)	220 (43%)	
Insurance			**<0.001**
Private	1440 (51%)	283 (55%)	
Medicaid	37 (1.3%)	65 (13%)	
Medicare	1352 (48%)	168 (33%)	
BMI	29 (26, 34)	32 (28, 37)	**<0.001**
Cardiomyopathy	385 (14%)	153 (30%)	**<0.001**
HTN	1731 (61%)	420 (81%)	**<0.001**
DM	455 (16%)	154 (30%)	**<0.001**
History of stroke	142 (5.0%)	54 (10%)	**<0.001**
History of TIA	67 (2.4%)	17 (3.3%)	0.216
VTE	169 (6.0%)	53 (10%)	**<0.001**
History of MI	71 (2.5%)	13 (2.5%)	0.99
OSA	720 (25%)	129 (25%)	0.829
Chronic lung disease	119 (4.2%)	32 (6.2%)	**0.045**
CKD	389 (14%)	88 (17%)	**0.048**
Abnormal liver function	10 (0.4%)	1 (0.2%)	>0.999
Alcohol use	45 (1.6%)	7 (1.4%)	0.693
AF class			0.162
Long‐standing persistent	16 (0.6%)	6 (1.2%)	
Paroxysmal	1750 (62%)	330 (64%)	
Persistent	1063 (38%)	180 (35%)	
Aflutter	669 (24%)	166 (32%)	**<0.001**
Prior CA	367 (13%)	25 (4.8%)	**<0.001**
Ablation type			0.237
Convergent	32 (1.1%)	2 (0.4%)	
Cryoablation	2591 (92%)	479 (93%)	
PFA	44 (1.6%)	11 (2.1%)	
RFA	162 (5.7%)	24 (4.7%)	
Composite complications	38 (1.3%)	13 (2.5%)	**0.045**
Cardiovascular	15 (0.5%)	7 (1.4%)	0.066
Systemic	2 (<0.1%)	0 (0%)	>0.999
GU/GI	2 (<0.1%)	0 (0%)	>0.999
Neurologic	6 (0.2%)	0 (0%)	0.599
Access site	9 (0.3%)	2 (0.4%)	0.682
Pulmonary	8 (0.3%)	5 (1.0%)	**0.038**
Discharge in NSR	2780 (98%)	507 (98%)	0.985
Prolonged LOS	120 (4.2%)	49 (9.5%)	**<0.001**

Abbreviations: CAD = coronary artery disease, CKD = chronic kidney disease, DM = diabetes mellitus, GU/GI = genitourinary, gastrointestinal, HTN = hypertension, LOS = length of stay, MI = myocardial infarction, NHB = non‐Hispanic Black, NHW = non‐Hispanic White, NSR = normal sinus rhythm, OSA = obstructive sleep apnea, PFA = pulsed field ablation, RFA = radiofrequency ablation, TIA = transient ischemic attack, VTE = venous thromboembolism.

^a^ Median (Q1, Q3); *n* (%).

^b^ Wilcoxon rank sum test; Pearson's Chi‐squared test; Fisher's exact test.

^c^ False discovery rate correction for multiple testing.

**TABLE 2:** Participant demographics by sex.

Characteristic	Male, *n* = 2162^a^	Female, *n* = 1183^a^	*p* value^b^
Age	66 (58, 73)	69 (62, 75)	**<0.001**
Race			**<0.001**
White	1866 (86%)	963 (81%)	
Black	296 (14%)	220 (19%)	
Insurance			**<0.001**
Private	1228 (57%)	495 (42%)	
Medicaid	53 (2.5%)	49 (4.1%)	
Medicare	881 (41%)	639 (54%)	
BMI	29 (26, 33)	30 (26, 36)	**0.006**
Cardiomyopathy	392 (18%)	146 (12%)	**<0.001**
HTN	1371 (63%)	780 (66%)	0.146
DM	404 (19%)	205 (17%)	0.331
History of stroke	113 (5.2%)	83 (7.0%)	**0.035**
History of TIA	41 (1.9%)	43 (3.6%)	**0.002**
VTE	132 (6.1%)	90 (7.6%)	0.095
History of MI	68 (3.1%)	16 (1.4%)	**0.002**
OSA	620 (29%)	229 (19%)	**<0.001**
Chronic lung disease	77 (3.6%)	74 (6.3%)	**<0.001**
CKD	218 (10%)	259 (22%)	**<0.001**
Abnormal liver function	5 (0.2%)	6 (0.5%)	0.212
Alcohol use	44 (2.0%)	8 (0.7%)	**0.002**
AF class			**<0.001**
Long‐standing persistent	16 (0.7%)	6 (0.5%)	
Paroxysmal	1290 (60%)	790 (67%)	
Persistent	856 (40%)	387 (33%)	
Aflutter	583 (27%)	252 (21%)	**<0.001**
Prior CA	273 (13%)	119 (10%)	**0.027**
Ablation type			0.81
Convergent	22 (1.0%)	12 (1.0%)	
Cryoablation	1981 (92%)	1089 (92%)	
PFA	39 (1.8%)	16 (1.4%)	
RFA	120 (5.6%)	66 (5.6%)	
Composite complications	30 (1.4%)	21 (1.8%)	0.382
Cardiovascular	13 (0.6%)	9 (0.8%)	0.585
Systemic	2 (<0.1%)	0 (0%)	0.543
GU/GI	1 (<0.1%)	1 (<0.1%)	>0.999
Neurologic	4 (0.2%)	2 (0.2%)	>0.999
Access site	5 (0.2%)	6 (0.5%)	0.212
Pulmonary	8 (0.4%)	5 (0.4%)	0.779
Discharge in NSR	2128 (98%)	1159 (98%)	0.334
Prolonged LOS	99 (4.6%)	70 (5.9%)	0.091

Abbreviations: CAD = coronary artery disease, CKD = chronic kidney disease, DM = diabetes mellitus, GU/GI = genitourinary, gastrointestinal, HTN = hypertension, LOS = length of stay, MI = myocardial infarction, NSR = normal sinus rhythm, OSA = obstructive sleep apnea, PFA = pulsed field ablation, RFA = radiofrequency ablation, TIA = transient ischemic attack, VTE = venous thromboembolism.

^a^ Median (Q1, Q3); *n* (%).

^b^ Wilcoxon rank sum test; Pearson's Chi‐squared test; Fisher's exact test.

^c^ False discovery rate correction for multiple testing.

Comorbidity burden differed significantly between race and sex in our study. NHB patients had a higher comorbidity burden compared to NHW patients, including cardiomyopathy (CM), hypertension (HTN), diabetes mellitus (DM), stroke, venous thromboembolism, and chronic kidney disease (CKD) (Table 1). Females had an increased number of a transient ischemic attack (TIA) and chronic lung disease, whereas males had higher numbers of CM and obstructive sleep apnea (Table 2). Males were more likely to have history of prior myocardial infarction compared to females. There was no significant difference in prior alcohol use disorder by race, but males were more likely to have a history of alcohol use disorder than females.

AF classification varied by sex, with females more likely to have paroxysmal AF, while persistent AF was more common in males. This variation in AF type was statistically significant by sex but not by race. NHB patients and males were more likely to have atrial flutter compared to NHW patients and females. There was a greater proportion of NHW patients and males that have received a prior CA compared to NHB patients and females. While complications were not significant by sex, NHB patients were more likely to have any complication and a pulmonary complication than NHW patients. There is no significant difference in ablation energy type or achieving sinus rhythm on discharge by race or sex.

In the multivariable logistic regression analysis, NHB race was independently associated with prolonged LOS (OR 1.9, 95% CI: 1.24–2.88, *p* = 0.003), after adjusting for key demographics, Medicaid status, cardiovascular and non‐cardiovascular comorbidities (including cardiomyopathy, diabetes, stroke, chronic lung disease, and chronic kidney disease), as well as procedural characteristics and complications (Table 3). Complications, convergent ablation, and certain comorbidities like CM, HTN, CKD, and abnormal liver function were also associated with prolonged LOS. There was no evidence of collinearity in the final models based on low variance inflation factor levels (Table S1). Given that complications were a sparse but strong predictor of prolonged LOS, a sensitivity analysis was performed which showed better model performance with the inclusion of complications (Figure S1).

**TABLE 3:** Combined univariable and multivariable regression analyses of factors associated with prolonged length of stay.

Covariate	OR^a^	95% CI^a^	*p* value
Univariable analysis			
Age	1.01	1.00, 1.03	0.077
BMI	1.03	1.01, 1.05	**<0.001**
NHB race	2.37	1.66, 3.33	**<0.001**
Female	1.31	0.95, 1.79	0.092
Medicaid	2.11	1.01, 3.94	**0.030**
Medicare	1.11	0.81, 1.51	0.5
Cardiomyopathy	2.17	1.52, 3.06	**<0.001**
Hypertension	2.20	1.52, 3.26	**<0.001**
Diabetes mellitus	1.74	1.21, 2.45	**0.002**
CVA	1.36	0.72, 2.36	0.3
TIA	2.03	0.89, 4.03	0.063
VTE	1.40	0.78, 2.34	0.2
History of MI	1.40	0.78, 2.34	0.2
OSA	1.21	0.85, 1.70	0.3
Chronic lung disease	2.92	1.71, 4.75	**<0.001**
CKD	2.46	1.72, 3.47	**<0.001**
Liver Disease	7.16	1.56, 25.0	**0.004**
Bleeding disorder	2.34	1.23, 4.11	**0.005**
Long standing persistent AF	1.89	0.30, 6.54	0.4
Paroxysmal AF	0.58	0.43, 0.80	**<0.001**
Persistent AF	1.68	1.23, 2.29	**0.001**
History of atrial flutter	1.52	1.09, 2.11	**0.012**
Prior CA	1.07	0.65, 1.68	0.8
Convergent ablation	11.0	5.17, 22.2	**<0.001**
Cryoablation	0.41	0.27, 0.63	**<0.001**
PFA	1.09	0.26, 2.99	0.9
RFA	1.58	0.86, 2.69	0.12
Any complications	135	65.6, 316	**<0.001**
Multivariable analysis			
BMI	1.04	1.01, 1.06	**0.002**
NHB race	1.90	1.24, 2.88	**0.003**
Cardiomyopathy	1.97	1.30, 2.94	**0.001**
Hypertension	1.60	1.03, 2.57	**0.042**
CKD	2.49	1.58, 3.85	**<0.001**
Liver disease	12.0	2.48, 44.3	**<0.001**
Convergent ablation	10.5	4.07, 27.3	**<0.001**
Complications	156	72.5, 377	**<0.001**

*Note*: Multivariable model adjusted for age, BMI, black race, female sex, Medicaid insurance, cardiomyopathy, diabetes, stroke, chronic lung disease, chronic kidney disease, atrial flutter, prior CA, convergent ablation, pulsed field ablation, radiofrequency ablation, and any complication.

Abbreviations: AF = atrial fibrillation, BMI = body mass index, CA = catheter ablation, CKD = chronic kidney disease, CVA = cerebrovascular accident, MI = myocardial infarction, NHB = non‐Hispanic black race, OSA = obstructive sleep apnea, PFA = pulse field ablation, RFA = radiofrequency ablation, TIA = transient ischemic attack, VTE = venous thromboembolism.

^a^ OR = odds ratio, CI = confidence Interval.

The mediation analysis found that the effect of race on LOS is partially mediated by a higher comorbidity burden (ACME = 0.007, *p* < 0.001), accounting for 17.8% of the effect, and obesity (ACME = 0.003, *p* = 0.012), accounting for 8.1% of the effect. Other candidate mediators including complications, Medicaid status, history of atrial flutter, and persistent AF did not show significant indirect effects (Table 4).

**TABLE 4:** Single mediator analysis of clinical and demographic factors assessing whether they mediate the association between non‐Hispanic Black (NHB) race and prolonged length of stay.

Mediator	Total effect	ACME (indirect effect)	ACME *p* value	ADE (direct effect)	ADE *p* value	Proportion mediated
Modified CCI	0.039	0.007	<0.001	0.032	0.002	0.178
Obesity	0.035	0.003	0.012	0.032	0.006	0.081
Complications	0.038	0.006	0.226	0.032	0.000	0.165
Medicaid	0.033	0.001	0.700	0.032	0.008	0.037
Atrial flutter	0.033	0.001	0.120	0.032	0.002	0.026
Persistent AF	0.031	−0.001	0.08	0.032	0.004	−0.027

*Note*: Each variable was tested in a separate mediation model to evaluate whether it served as a mediator in the relationship between NHB race and prolonged length of stay. The total effect of NHB race was decomposed into the average causal mediation effect (ACME, indirect effect) and the average direct effect (ADE). The proportion mediated reflects the percentage of the total effect explained by the indirect path through each mediator.

Abbreviations: ACME = average causal mediation effect, ADE = average direct effect; AF = atrial fibrillation; CCI = Modified Charlson comorbidity index, NHB = non‐Hispanic Black.

## 4 Discussion

We sought to investigate race and sex differences in patient and procedural characteristics and their impact on LOS after CA for AF. In our cohort, there were important differences in baseline characteristics including a high comorbidity burden among NHB and female patients undergoing CA compared to NHW patients. We found no significant association of sex with CA. Although clinical factors such as comorbidities and procedural factors like ablation type (e.g., convergent ablation) and complications were linked to prolonged LOS, NHB patients had an independently higher likelihood that was not fully mediated by complications or other factors. Our results highlight the important potential effect of non‐procedural factors on prolonged LOS.

Previous literature has consistently identified factors such as advanced age, frailty, comorbidities, complex ablations, and procedures in low‐volume hospitals as predictors of prolonged LOS following CA [16, 17]. To our knowledge, our findings are the first to identify race as an independent predictor of LOS after CA. As stated above, NHB patients were found to have a higher burden of comorbidities compared to NHW patients, which has been associated with increased complications and prolonged LOS [17]. Comorbidities escalate the complexity of postoperative management, thus increasing the chances of complications such as bleeding, infection, thromboembolism, or death from cardiovascular events [18]. Additionally, comorbidities can lead to slower recovery rates, further prolonging LOS [18]. Our mediation analysis revealed that the prolonged LOS among NHB was partially mediated by a higher comorbidity burden and obesity, but there remained a significant direct effect of race on the outcome.

Our findings highlight a dual disparity: not only do NHB patients present with a greater burden of cardiovascular and non‐cardiovascular comorbidities at the time of ablation, but race also remains independently associated with prolonged LOS even after adjusting for these differences. This suggests that NHB patients may be accessing care later in the disease process, possibly due to systemic barriers such as delayed referrals or limited access to specialty care [19]. Similar differences in access have been noted in other areas of cardiovascular procedural care, with NHB patients being less likely to undergo cardiac catheterization following an acute MI, cardiac resynchronization therapy in HFrEF, and other invasive management [20, 21]. While comorbidities likely contribute to prolonged hospitalization, the persistence of race as an independent predictor in our adjusted model highlights the need to examine factors beyond clinical risk alone. These may include factors that are challenging to measure such as inequities in pre‐ and post‐procedural support, communication gaps, or implicit bias [22]. Other important factors include health literacy, socioeconomic status, psychosocial factors, and systemic factors like hospital environment (i.e., staffing and resources) that delay treatment and prolong hospital LOS [22]. Understanding and addressing these upstream determinants is essential to ensure equitable care and outcomes for all patients undergoing ablation.

While sex was not associated with prolonged LOS, our study identified key differences between men and women in AF classification and comorbidity burden. Female patients were more likely to be older, have paroxysmal AF, TIAs, and chronic lung disease compared to males. Similar findings were noted in the Catheter Ablation versus Antiarrhythmic Drug Therapy for Atrial Fibrillation (CABANA) trial, which found that women undergoing a CA procedure were older, had a higher burden of disease associated with AF, and were more likely to have paroxysmal AF [23, 24]. These differences may be explained by the variation in presentation among sexes, with women presenting with more atypical symptoms that can easily be mistaken for other medical conditions, leading to delayed diagnosis and subsequent treatment [20, 24]. Age at diagnosis is an important prognostic indicator of a successful procedure, with a follow‐up of the CABANA trial concluding that the largest benefit of CA is seen in younger patients [25].

Prolonged LOS is often used as a quality metric to evaluate hospital efficiency and patient outcomes [26]. However, our findings suggest that this metric should also consider the effect of non‐procedural factors, such socioeconomic barriers, disproportionate access to cardiovascular care, and interpersonal relationships between patients and providers [18, 19]. For example, incorporating ZIP Code‐level SDOH in the Get With The Guidelines‐Heart Failure registry showed that these social determinants were independently associated with LOS after adjusting for clinical status and hospital‐level factors [27]. Adjusting for SDOH may help refine this quality metric and help target resources toward addressing non‐procedural factors associated with prolonged LOS in order to improve quality of care.

While our findings are the first to identify race as an independent predictor of LOS after CA, our study has several important limitations. The “residual direct effect” approach, which estimates race disparity as the remaining difference between groups after adjusting for other covariates, has certain constraints. The independent effect of race on our primary outcomes is challenging to interpret and likely reflects interactions and mediation effects between race and unmeasured confounders like implicit bias, patient‐provider rapport and trust, health literacy and patient preference among others. Data scarcity did not allow us to adjust for important covariates known to affect LOS and outcomes such as social determinants of health. It is unclear whether differences can be attributed to unfair differences in care delivery due to bias or other factors that may mediate the effect of race, such as socioeconomic status. Other important clinical markers of disease severity like left ventricular ejection fraction and left atrial volume were missing, limiting our ability to fully adjust for health status.

Selection bias is also possible due to exclusion of patients with missing data, however, the number excluded was relatively small. Although the sample sizes of the racial groups were imbalanced, the proportion of NHB individuals in our study (15.4%) closely reflects their representation in the US population (14.4%), supporting the generalizability of our findings. Furthermore, there was a sufficient number of outcome events in both racial groups to allow for a meaningful analysis. While our mediation analysis showed no significant mediation effect of complications between race and prolonged LOS, it is possible that the sparsity of this outcome and unmeasured confounders may influence its relationship with both race and LOS. Future studies incorporating more granular data on these variables and larger sample sizes may clarify these pathways.

Despite these limitations, our study provides contributions to the existing literature on inequities in AF care for NHB patients using real‐world data on this understudied population. While the effects of SDOH have been well documented in other disease areas like ischemic heart disease and heart failure, only minor attention has been paid to their impact on AF care. This gap in the literature was the focus of an expert workshop and report by The National Heart, Lung and Blood Institute that called for more intensive research efforts to address inequities [28]. Our findings identify important priorities for data collection on SDOH in future iterations of national AF registries such as the NCDR database to transform and improve equity in AF research.

## 5 Conclusions

Among patients undergoing CA for AF, there were key differences in comorbidity burden by race and sex and significantly increased odds of prolonged LOS among NHB patients. The association of race and LOS was partially mediated by a higher comorbidity burden and obesity among NHB patients, but a significant residual direct effect of race remained that is likely a proxy for inequities and structural biases not captured by current data sources. These results suggest that attempts to reduce LOS after CA may need to account for the role of upstream non‐procedural factors like social determinants of health in prolonging LOS after CA for AF.

## Disclosure

The authors have nothing to report.

## Supporting information




**Supporting Table S1**: Variance inflation factors (VIFs) were calculated to assess multicollinearity among covariates included in the multivariable regression model.
Figure 1. Sensitivity analysis performed with and without the composite variable of complications. Receiver operator curve (ROC) and area under curve (AUC) for the models fitted with and without this composite variable are shown.
