# Supplementary material for: Racial Differences in Length of Stay After Atrial Fibrillation Ablation
Source: Pacing Clin Electrophysiol. 2025 Aug 21;48(10):1148–56. doi: 10.1111/pace.70029 (PMC12504919; doi:10.1111/pace.70029)

Supplementary Materials

Table 1. Variance inflation factors (VIFs) were calculated to assess multicollinearity among covariates included in the multivariable regression model

Figure 1. Sensitivity analysis performed with and without the composite variable of complications. Receiver operator curve (ROC) and area under curve (AUC) for the models fitted with and without this composite variable are shown.

**Table 1**. Variance inflation factors (VIFs) were calculated to assess multicollinearity among covariates included in the multivariable regression model.

| Covariate | VIF |
| --- | --- |
| BMI | 1.19 |
| NHB Race | 1.11 |
| Cardiomyopathy | 1.07 |
| Hypertension | 1.08 |
| Chronic lung disease | 1.01 |
| CKD | 1.16 |
| Liver Disease | 1.01 |
| Persistent AF | 1.05 |
| Convergent Ablation | 1.49 |
| Cryo Ablation | 1.47 |
| Any Complication | 1.03 |

**Figure 1**. Sensitivity analysis performed with and without the composite variable of complications. Receiver operator curve (ROC) and area under curve (AUC) for the models fitted with and without this composite variable are shown.


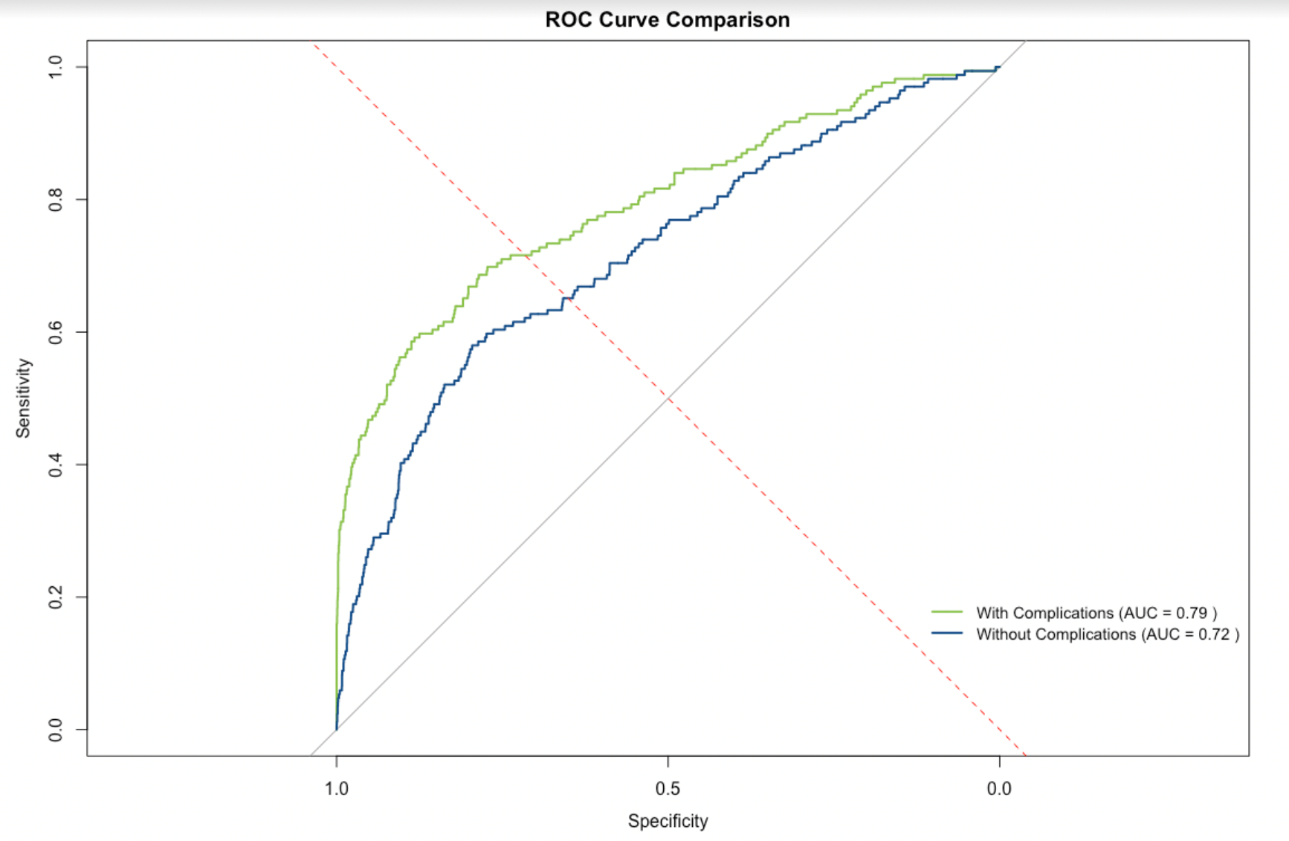

Supplement: Supplementary file 1 — Supporting Table S1: Variance inflation factors (VIFs) were calculated to assess multicollinearity among covariates included in the multivariable regression model. Figure 1. Sensitivity analysis performed with and without the composite variable of complications. Receiver operator curve (ROC) and area under curve (AUC) for the models fitted with and without this composite variable are shown. [file PACE-48-1148-s001.docx]
